# Proof-of-principle 4-marker spatial profiling reveals distinct, location-independent immune clusters in biliary tract cancers

**DOI:** 10.3389/pore.2026.1612389

**Published:** 2026-05-28

**Authors:** Federico Rossari, Michele Sala, Margherita Rimini, Silvia Camera, Mara Persano, Giovanni Tonon, Chiara Bonini, Maria Giulia Cangi, Lorenza Pecciarini, Claudio Doglioni, Maurilio Ponzoni, Chiara Balestrieri, Federica Pedica, Andrea Casadei-Gardini

**Affiliations:** 1 Department of Oncology, IRCCS San Raffaele Scientific Institute, Milan, Italy; 2 “Vita-Salute” San Raffaele University, Milan, Italy; 3 Center for Omics Sciences, IRCCS San Raffaele Scientific Institute, Milan, Italy; 4 University of Milan, Milan, Italy; 5 Politecnico di Milano, Milan, Italy; 6 Experimental Hematology Unit, Division of Immunology, Transplantation and Infectious Diseases, IRCCS San Raffaele Scientific Institute, Milan, Italy; 7 Pathology Unit, IRCCS San Raffaele Scientific Institute, Milan, Italy

**Keywords:** biliary tract cancer, cholangiocarcinoma, immunofluorescence, immunotherapy, tumor microenvironment

## Abstract

Biliary tract cancers (BTCs) are aggressive malignancies with limited treatment options. Despite advances with durvalumab plus chemotherapy, prognosis remains poor. The tumor immune microenvironment (TIME) is increasingly recognized as a determinant of immunotherapy response, yet its spatial organization in BTC is poorly understood. We applied multiplex immunofluorescence to pre-treatment tumor samples from 11 patients with advanced BTC, staining for CD4, CD8, CD20, and CD163 to identify T cells, B cells, and macrophages. Across 198 regions of interest sampled at the invasive tumor margin, approximately 400,000 individual cells were segmented and phenotyped. Spatial organization was analyzed using hierarchical clustering, distance-based approaches, and cellular neighborhood classification. Unsupervised analysis identified two distinct immune phenotypes, one enriched in CD4^+^ T cells and CD163^+^ macrophages and the other in CD8^+^ T cells and CD20^+^ B cells. These phenotypes displayed distinct spatial architectures, with CD4^+^ T cells and CD20^+^ B cells acting as organizing hubs, respectively. Targeted sequencing, available for a subset of cases, showed a higher frequency of co-occurring TP53 and ARID1A alterations in the B-cell–enriched phenotype. No statistically significant differences in overall or progression-free survival were observed between immune phenotypes. However, among patients treated with adjuvant cisplatin/gemcitabine alone, lower CD20/CD8 fractions and higher CD163 abundance at baseline were associated with improved survival. Spatial immune profiling revealed distinct BTC immune architectures at the invasive margin, with different possible impact on survival, thus warranting further characterization and validation in larger cohorts.

## Introduction

Biliary tract cancers (BTCs) are a heterogeneous group of malignancies arising from the epithelial lining of the biliary tree, including intrahepatic (iCCA), perihilar (pCCA), distal (dCCA) cholangiocarcinoma, and gallbladder cancer (GBC). Although individually rare, together they account for approximately 3% of all gastrointestinal cancers, with a rising incidence observed globally, particularly in regions such as Southeast Asia and South America [1, 2]. Prognosis remains dismal, with 5-year overall survival rates below 10% in advanced-stage disease [3]. This poor prognosis is largely due to late diagnosis, limited resectability, and resistance to systemic therapy.

The current first-line therapeutic regimen for advanced BTC is cisplatin combined with gemcitabine (CG) and immunotherapy. In fact, the addition of durvalumab, an anti–Programmed Cell Death Ligand 1 (PD-L1) immune checkpoint inhibitor, to CG (forming the CGD regimen) has recently showed a modest but significant improvement in overall survival (OS) in the TOPAZ-1 trial, leading to its adoption as a new standard of care [4]. These results were also consistent with findings from real-world studies [5]. Similarly, the anti-Programmed Cell Death 1 (PD-1) pembrolizumab has been approved in combination with CG in the same therapeutic setting following the positive OS results of the KEYNOTE-966 trial [6]. However, the survival benefit of these regimens was not uniform, and predictive biomarkers for selecting patients who will benefit from chemoimmunotherapy remain poorly defined.

Understanding the tumor immune microenvironment (TIME) can foster optimization of immunotherapy strategies. BTCs are typically considered “immune-excluded” or “cold” tumors [7, 8], yet emerging evidence suggests substantial heterogeneity in immune infiltration and spatial architecture across BTC subtypes [9–11]. Studies have identified the presence of tumor-infiltrating lymphocytes (TILs), tumor-associated macrophages (TAMs), and B cells in the BTC microenvironment, with varying associations with prognosis [12–14]. However, most analyses to date have relied on bulk RNA-sequencing or conventional immunohistochemistry, which do not allow precise and detailed spatial information, critical for understanding immune–tumor and immune–immune interactions [10, 14].

Recent advances in spatial biology, including multiplex immunofluorescence (mIF), allow for the simultaneous detection of multiple immune lineages within intact tissue architecture [15]. These technologies provide single-cell resolution of immune architecture, enabling analysis of cell–cell proximity, microanatomical neighborhoods, and tissue organization, features increasingly recognized as critical determinants of tumor progression and therapeutic response [16]. Evidence from other solid tumors, including breast and colorectal cancers [17–19], confirms that spatial analyses can uncover clinically relevant immune architectures. By contrast, comparable studies in BTC remain scarce, despite the urgent need for immune biomarkers in this disease.

In this proof-of-principle study, we applied Akoya Opal multiplex immunofluorescence (TSA-mIF) to characterize four major immune lineages, CD4^+^ T cells, CD8^+^ T cells, CD20^+^ B cells, and CD163+ macrophages, in pre-treatment BTC samples. Our objective was to determine whether distinct immune clusters could be identified based on lineage abundance and spatial distribution, and whether these correlate with anatomical site, mutational status, or clinical outcomes. Given the very limited literature on spatial immune profiling in BTC, we hypothesized that such analyses may reveal clinically relevant ecosystems in this disease and provide a foundation for larger, hypothesis-driven studies integrating spatial immuno-oncology into BTC research.

## Materials and methods

### Study cohort and sample selection

This study included 11 treatment-naïve patients with biliary tract adenocarcinoma, representing a spectrum of anatomical subtypes: iCCA (n = 4), pCCA (n = 5), dCCA (n = 1), and GBC (n = 1). Formalin-fixed, paraffin-embedded (FFPE) tumor samples were obtained from surgical resections prior to the initiation of systemic therapy. Molecular profiling was performed on available FFPE samples using next-generation sequencing (NGS). The FoundationOne CDx assay, covering 324 genes and providing tumor mutational burden (TMB) and microsatellite stability status, was used for patients P01, P02, P03, P05, P07, P09, P10 and P11. Patient P06 was analyzed using the Myriapod NGS Cancer Panel, covering 56 genes and providing microsatellite stability status, with orthogonal validation by fluorescence *in situ* hybridization (FISH). No molecular analysis was available for P04 and P08.

Clinical data were collected retrospectively. All patients received first-line systemic treatment for advanced disease, consisting of cisplatin and gemcitabine with or without durvalumab (CGD or CG, respectively), according to clinical eligibility and regimen availability at the time of treatment. Overall survival (OS) was defined as the time from initiation of first-line therapy to death from any cause, while progression-free survival (PFS) was defined as the time from treatment initiation to disease progression or death. Treatment response was assessed according to RECIST (version 1.1) criteria. Patients achieving complete response (CR) or partial response (PR) were classified as responders (R), whereas those with stable disease (SD) or progressive disease (PD) were classified as non-responders (NR).

Baseline clinical and genomic characteristics are reported in Table 1, while follow-up and outcome data are summarized in Table 2.

**TABLE 1:** Individual clinical and genomic characteristics of the study population.

Patient	Sex	Age	Location	TNM at diagnosis	Genotype	TMB^§^
P01	F	72	pCCA	pT1a, pN0, G2	***CDKN2A*** Y44fs*1, ***ARID1A*** Y551fs*72, ***BRAF*** K601E; MSS	0.00
P02	F	59	iCCA	mpT2, pN1, G2	***NTRK1*** amp#, ***MCL1*** amp#, ***MYC*** amp#/rearr, ***FGFR2*** rearr, ***NFE2L2*** L30H; MSS	1.26
P03	M	77	pCCA	pT2a, pN0, G2	***KDM5A*** amp#, ***ARID1A*** trunc, ***KDM6A*** dup, ***PIK3R1*** R514C, ***U2AF1*** S34F, ***TP53*** R213L; MSS	7.57
P04	M	68	pCCA	PT3, pN2, G3	N/A	N/A
P05	M	68	pCCA	pT2a, pN0, G3	***ARID1A*** S2269* (sub), ***ACVR1B*** R456*, ***CCNE1*** amp, ***EZH2*** del, ***FGFR3*** amp, ***PIK3CA*** E542K/E545K (sub), ***RBM10*** splice 2167-1G>A, ***SMAD4*** E330K, ***TP53*** G245S; MSS	10.09
P06	F	71	pCCA	pT3, pN0, G2	***KRAS*** G12D, ***FGFR2*** rearr; MSS	N/A
P07	M	70	iCCA	pT4, pN0, G2	***BRCA2*** W1692fs*3, ***KRAS*** Q61H, ***RBM10*** splice 1951-1G>C; MSS	N/A
P08	M	80	iCCA	pT4, pN1, G3	N/A	N/A
P09	M	77	dCCA	pT2, pN1, G3	***FGFR3*** amp#, ***GATA6*** amp, ***ASXL1*** E635fs*15 (sub), ***APC*** T1556fs*3, ***PIK3CA*** E545K, ***JAK2*** V617F (sub), ***TP53*** E171fs*3; MSS	5.04
P10	M	54	iCCA	PT4, pN1, G3	***CDKN2A*** loss, ***CDKN2B*** loss, ***DNMT3A*** Y683*, ***KRAS*** G12V; MSS	1.26
P11	M	59	GBC	pT2b, G2	***ARID1A*** D1912fs*5, ***KDM6A*** loss, ***PIK3CA*** H1047R, ***TP53*** C277F, ***VHL*** R210W; MSS	1.26

The demographic, clinico-pathological, and targeted NGS, findings of the cohort are reported.

Cancer-related genes are indicated in bold.

Abbreviations and symbols: F, female; M, male; pCCA, perihilar cholangiocarcinoma; dCCA, distal cholangiocarcinoma; iCCA, intrahepatic cholangiocarcinoma; GBC, gallbladder cancer; MSS, microsatellite stable; amp, amplification; rearr, rearrangement; del, deletion; trunc, truncation; dup, duplication; sub, subclonal; (§) mutations/Mb; (#) equivocal copy number; N/A, not available.

**TABLE 2:** Individual clinical outcomes and treatment respons**e**.

Patient ID	Treatment	Best Response	PFS (months)	OS (months)	Responder	Status
P01	CG	SD	4.2	24.1	NR	Deceased
P02	CG	PD	2.4	23.9	NR	Alive
P03	CG	SD	2.9	38	NR	Alive
P04	CG	PD	3.2	13.5	NR	Alive
P05	CGD	CR	33	33	R	Deceased
P06	CGD	PR	30	30	R	Deceased
P07	CGD	PR	16	27	R	Alive
P08	CG	SD	9.5	9.5	NR	Deceased
P09	CG	PD	3.2	11.6	NR	Alive
P10	CG	PD	1.5	1.5	NR	Deceased
P11	CGD	SD	5	11.7	NR	Deceased

Clinical outcomes of systemic treatments after diagnosis of advanced-stage disease (*de novo* or relapsed). Abbreviations: CG, cisplatin plus gemcitabine; CGD, CG, plus durvalumab; SD, stable disease; PD, progressive disease; PR, partial response; CR, complete response; PFS, progression free survival; OS, overall survival; R, responder; NR, non-responder.

### Multiplex immunofluorescence staining and imaging

We tested a panel of 4 antibodies using multiplex immunofluorescence (mIF) staining using a commercially available kit (Opal 6- plex Detection Kit, catalogue number NEL811001KT, Akoya Biosciences Inc., Marlborough, MA, USA) with a validated antibody panel: CD4 (Opal 480 MSI), CD8 (Opal 520 Cy5 MSI), CD163 (Opal 620 Texas Red), and CD20 (Opal 488, FITC), with DAPI (blue) as nuclear counterstain. The tissue slides underwent a sequence of deparaffinization cycles (5 min each), then antigen retrieval was performed at 97 °C with using BOND Epitope Retrieval Solution 2 (Leica), primary antibody (45 min at 37 °C), incubation with multilinked HRPpolymer, incubation with fluorochrome-tyramid complex (Akoya Opal® Polaris 7-Color IHC Kit, NEL861001KT) (20 min at room temperature) and stripping of antibody complexes with ER2 solution (20 min at 97 °C), before the next immunolabeling cycle. After the last immuno-labeling cycle, spectral DAPI (Akoya Biosciences) was added to the slides for 5 min as a nuclear counterstain, and coverslips were mounted with ProLongTM Diamond Antifade Mountant (InvitrogenTM, ThermoFisher Scientific) mounting medium. All the steps previously described, except for the coverslips mounting, were performed on Leica Bond RX. Images were acquired using the Akoya PhenoImager HT 2.0 platform, and spectral unmixing was performed using the built-in synthetic spectra library to generate unmixed images for downstream analysis.

### Image analysis and cell phenotyping

Digital image analysis was performed using QuPath (v0.5.1) [20] for single-cell segmentation and classification based on fluorescence intensity and localization. Based on the pathologist’s evaluation of H&E slide as a representative part of the whole tumor and multiple immunofluorescence analysis, an initial set of 25 candidate regions of interest (ROIs) per patient (500 μm in diameter) was manually selected to accurately target the invasive margin. Quality control filtering excluded regions with imaging artifacts or >60% DAPI-only cells. This threshold was applied to retain regions containing enough phenotyped cells for reliable spatial neighborhood analysis, while excluding areas that could not be robustly characterized with the current marker panel. In total, 198 ROIs were analyzed across the cohort corresponding to 15–21 representative ROIs per patient. Individual cells were segmented using StarDist algorithm [21] and annotated based on lineage-specific marker expression after supervised thresholding and classifier tuning. Across all ROIs, 397,270 single cells were segmented and annotated. Data were exported at single-cell and ROI levels for downstream analysis in a custom Python pipeline. Further details on the computational pipeline are provided in the *Data and code availability statement*.

### Cell type analysis and immune clustering

For each patient, we pooled single-cell phenotype labels across all ROIs and computed the relative fraction of each cell phenotype (CD4^+^, CD8^+^, CD20^+^, CD163^+^ and DAPI-only). Immune cell fractions were retained as patient features for clustering analysis. To stabilize variance and normalize feature scales, fractions were logit-transformed and standardized to zero mean and unit variance. Patients were then grouped by unsupervised agglomerative clustering using Euclidean distance and Ward’s linkage (cophenetic correlation = 0.617). The optimal number of clusters was determined by dendrogram analysis. Given the non-normal distribution of cell fractions (Shapiro-Wilk test p < 0.05), differences between clusters were assessed using Mann-Whitney U tests with Benjamini-Hochberg correction and significance at p < 0.05.

### Cellular neighborhood analysis

Beyond composition, we explored the spatial organization of the TIME by characterizing local cellular microenvironments through cellular neighborhood (CN) analysis. Each cell was assigned to a neighborhood defined by the composition of its 10 nearest neighbors. Local environments were clustered with the k-means++ algorithm. After testing multiple values of k, k = 8 was selected as the biologically optimal configuration, yielding eight CNs (CN0–CN7) with distinct immune cell enrichments. For spatial visualization of CNs within ROIs, Voronoi diagrams were constructed using methods adapted from [22]. Due to non-normal CN distributions (Shapiro-Wilk p < 0.05), inter-cluster differences were tested using Mann-Whitney U tests and Benjamini-Hochberg correction (p < 0.05).

### Proximity analyses

We investigated enrichment and co-localization patterns between immune lineages. First, we assessed the local co-occurrence of immune cell types through a neighborhood enrichment analysis [23], measuring, for each immune lineage serving as reference, the enrichment of each phenotype around its cells. Each lineage was considered in turn as a reference to capture pairwise interaction patterns across all cell types. Enrichment scores were computed and plotted for each cluster separately, using *squidpy*’s *nhood_enrichment()* functions, which evaluate statistical significance through permutation-based testing, setting a 50 μm radius and n = 1,000 iterations.

Finally, proximity analysis was conducted using a custom-developed Python algorithm to quantify spatial co-occurrence patterns between immune cell types within variable radii (from 10 to 200 μm at 10 μm intervals). For each ROI, the algorithm computed pairwise distance matrices between all cells using Euclidean coordinates. Cell pairs within each distance threshold were identified, and co-occurrence frequencies were calculated as the proportion of each cell type found in proximity to every other cell type, normalized by row to obtain relative fractions. ROI-level proximity data were then aggregated by computing mean and standard deviation across ROIs within each patient cluster.

### Haematoxylin-eosin evaluation of tumor slides

We blindly reviewed the H&E slides of the tumor to describe the immune microenvironment at the invasive margin, subdividing between those that showed lymphoid aggregates in proximity of the tumor and those that presented only loose inflammatory infiltration. The inflammatory population in the portal tracts in the background liver parenchyma was not considered for the analysis.

### Statistical and survival analysis

Categorical variables were summarized descriptively, and comparisons between groups were performed using Fisher exact test or Chi-square test as specified in Supplementary Table S1. Survival outcomes were estimated using the Kaplan-Meier method, with OS and PFS compared between groups using the log-rank test. Median survival and 95% confidence intervals were reported, and hazard ratios (HRs) were estimated using univariate Cox proportional hazards models.

Given the limited sample size and number of events, all survival analyses were considered exploratory and hypothesis-generating.

## Results

### Clinical information and analysis workflow

To profile the spatial immune architecture of BTC prior to systemic therapy, we analyzed tumor samples from 11 treatment-naïve patients (4 iCCA, 5 pCCA, 1 dCCA, and 1 GBC). Baseline demographic and clinical characteristics, including sex, age, TNM classification, and mutational status, are reported in Table 1.

**FIGURE 1:**
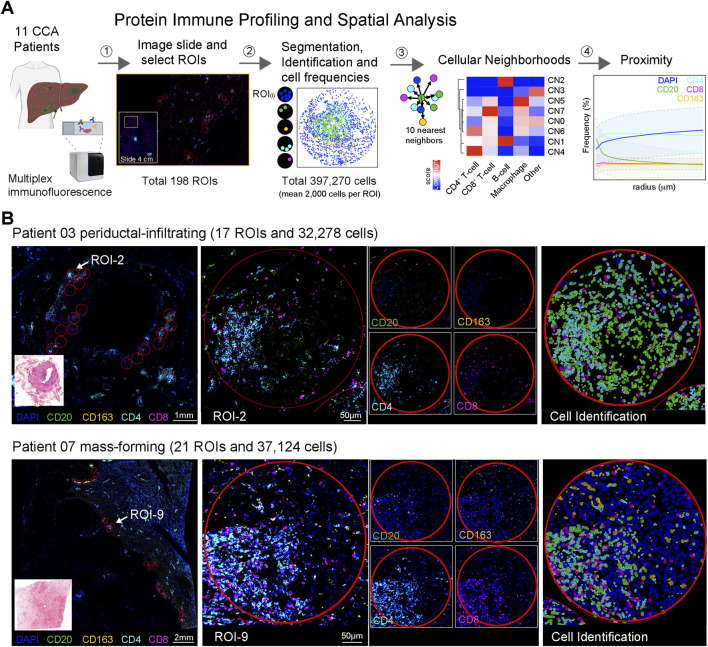
Analytical workflow and representative tissue imaging. **(A)** Schematic overview of the analytical workflow, from Opal mIF image acquisition to computational processing and downstream analyses. **(B)** mIF images from two representative patients with periductal infiltrating (Patient 3) and mass-forming (Patient 7) invasive patterns. Cell nuclei (DAPI) and CD4, CD8, CD20, and CD163 signals are displayed in pseudocolors. Panels present whole-slide overviews with selected ROIs (500 μm in diameter and ∼2,000 cells on average), with H&E images as insets. Individual marker channels and final cell identification are shown for single representative ROIs. Scale bars are indicated in the figure.

The analytical workflow, from image acquisition to spatial modeling, is illustrated in Figure 1A (see Methods for details). Briefly, sequential H&E-stained sections were reviewed by a pathologist to classify tumors by growth pattern, yielding four mass-forming (Patients 2, 6, 7, and 10) and seven periductal-infiltrating cases (Patients 1, 3, 4, 5, 8, 9, and 11), and to identify the invasive tumor margins (Supplementary Figure 1A).

Following this morphological guidance, whole-slide mIF scans were acquired and processed for fluorescence alignment. To capture the heterogeneity of the tumor-immune interface, we selected 25 representative ROIs per patient at the invasive margin. After quality filtering, 198 ROIs were retained, and approximately 400,000 individual cells were annotated. Representative tissue sections and ROIs are shown in Figure 1B; Supplementary Figure 2A.

Downstream analyses were performed to quantify immune lineage abundance, perform unsupervised clustering, and reconstruct spatial cellular neighborhoods at the invasive margin.

### Spatial immune profiling reveals two distinct clusters in BTC

**FIGURE 2:**
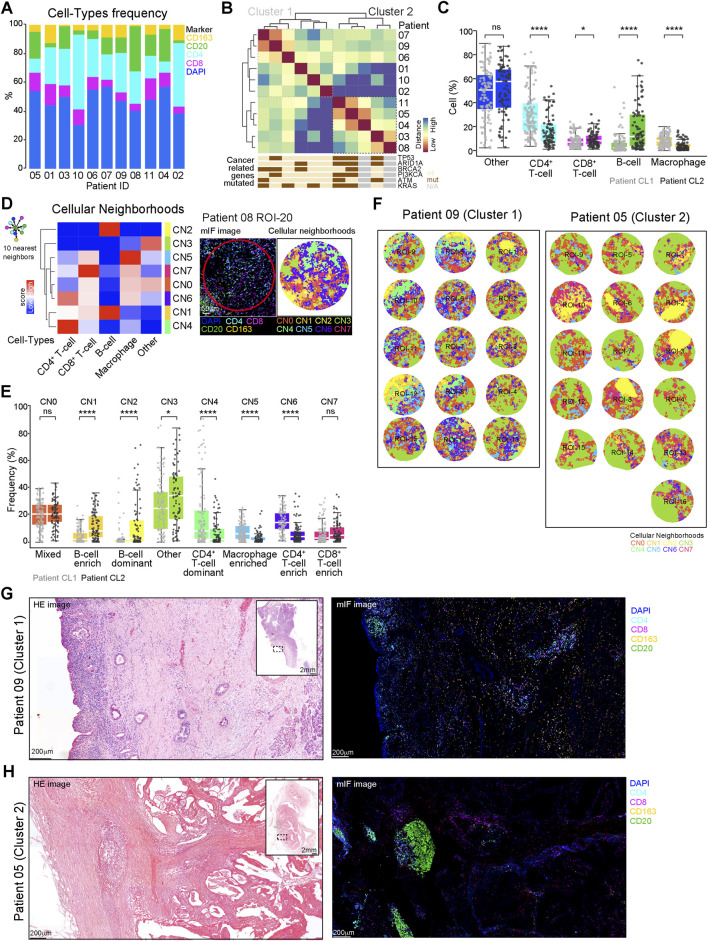
Immune clustering and cellular neighborhood analysis of BTC samples. **(A)** Relative abundance of CD4^+^ T cells, CD8^+^ T cells, CD20^+^ B cells, and CD163^+^ macrophages and DAPI-only cells across 11 BTC patients. **(B)** Unsupervised hierarchical clustering of patients based on immune cell fractions (Euclidean distance, Ward’s linkage). Heatmap displays pairwise distances between patients, revealing two distinct clusters. Mutational status for TP53, ARID1A, and PI3KCA from NGS analysis is also indicated. **(C)** Boxplots showing immune lineage fractions stratified by patient cluster. Each dot represents the relative fraction of a specific cell type within an individual ROI. **(D)** Cellular neighborhoods (CN0–CN7) defined by clustering cells based on their 10 nearest neighbors. Left panel heatmap displays immune composition signatures characterizing each CN. Right panels demonstrate spatial CN assignment visualized through Voronoi tessellation in a representative ROI. **(E)** Boxplots comparing CN distributions between clusters. Each dot represents the relative fraction of a specific CN within an individual ROI. **(F)** Visualization of all ROIs from two representative patients, Patient 9 (CL-1) and Patient 5 (CL-2), displayed as Voronoi diagrams colored by CN assignment. **(G,H)** H&E and mIF images of two representative patients, Patient 9 (CL-1) and Patient 5 (CL-2), showing immune aggregates in proximity to the tumor. Cell nuclei (DAPI) and CD4, CD8, CD20, and CD163 signals are displayed in pseudocolor. Scale bars are indicated in the figure. Mann-Whitney U tests with Benjamini-Hochberg correction were used for comparisons. Asterisks indicate significance levels (ns p > 0.05, *p ≤ 0.05 and ****p ≤ 0.0001).

We first quantified the relative abundance of CD4^+^ T cells, CD8^+^ T cells, CD20^+^ B cells, CD163^+^ macrophages across patients, revealing marked inter-patient variability in immune composition (Figure 2A). To investigate whether patients could be grouped according to their immune profiles, we applied bottom-up hierarchical clustering to immune cell type fractions using Euclidean distance and Ward linkage (Figure 2B). This analysis separated the cohort into two major clusters: Cluster 1 (CL-1, n = 6 patients) and Cluster 2 (CL-2, n = 5 patients). We then compared immune composition between clusters. CL-1 was enriched in CD4^+^ T cells and CD163^+^ macrophages (Mann-Whitney U test, p < 0.0001 and p < 0.0001, respectively), whereas CL-2 was characterized by higher proportions of CD8^+^ T cells and CD20^+^ B cells (p = 0.025 and p < 0.0001, respectively) (Figure 2C).

We next asked whether compositional differences were paralleled by distinct spatial organizations. To test this, we applied cellular neighborhood (CN) analysis, in which each cell was assigned to a neighborhood defined by the phenotypic composition of its ten nearest neighbors. Across all samples, we identified eight discrete CNs (CN0–CN7), each representing distinct patterns of local cell interactions (Figure 2D, left). The defining characteristics of these niches included: B-cell dominance (CN1 and CN2, with CN1 also showing a CD4^+^/CD8^+^ T-cell component), CD4^+^ T-cell enrichment (CN4 and CN6), and macrophage or CD8^+^ T-cell prevalence (CN5 and CN7, respectively), while CN0 and CN3 displayed more heterogeneous compositions without a dominant lineage. A representative ROI is shown with Voronoi tessellation illustrating CN assignment (Figure 2D, right).

We then compared CN distribution across clusters. Patients in CL-2 exhibited a higher prevalence of CD20^+^ B cells that frequently co-localized with CD8^+^ T cells (CN1 and CN2), suggestive of tertiary lymphoid structure (TLS)-like arrangements (Mann-Whitney U test, p < 0.0001, <0.0001 and 0.032, respectively). In contrast, CL-1 was enriched in regions dominated by CD4^+^ T cells and CD163^+^ macrophages with limited cytotoxic infiltration (CN4, CN5 and CN6; all p < 0.0001) (Figure 2E). We displayed CN assignments in two representative patients using Voronoi diagrams for each cluster: Patient 9 (CL-1) and Patient 5 (CL-2) (Figure 2F). Comprehensive CN maps for all other patients are shown in Supplementary Figure 3.

Although the comparison was performed on a limited number of cases, all tumors lacking TLS-like aggregates and showing only mild or loosely organized inflammatory infiltrates on H&E evaluation belonged to CL-1 (see representative example in Figure 2G). In contrast, tumors displaying an inflammatory infiltrate organized in TLS-like structures were predominantly associated with the CL-2 phenotype (4 of 5 cases; see representative example in Figure 2H).

### Proximity networks reveal divergent immune interactions in immune clusters

**FIGURE 3:**
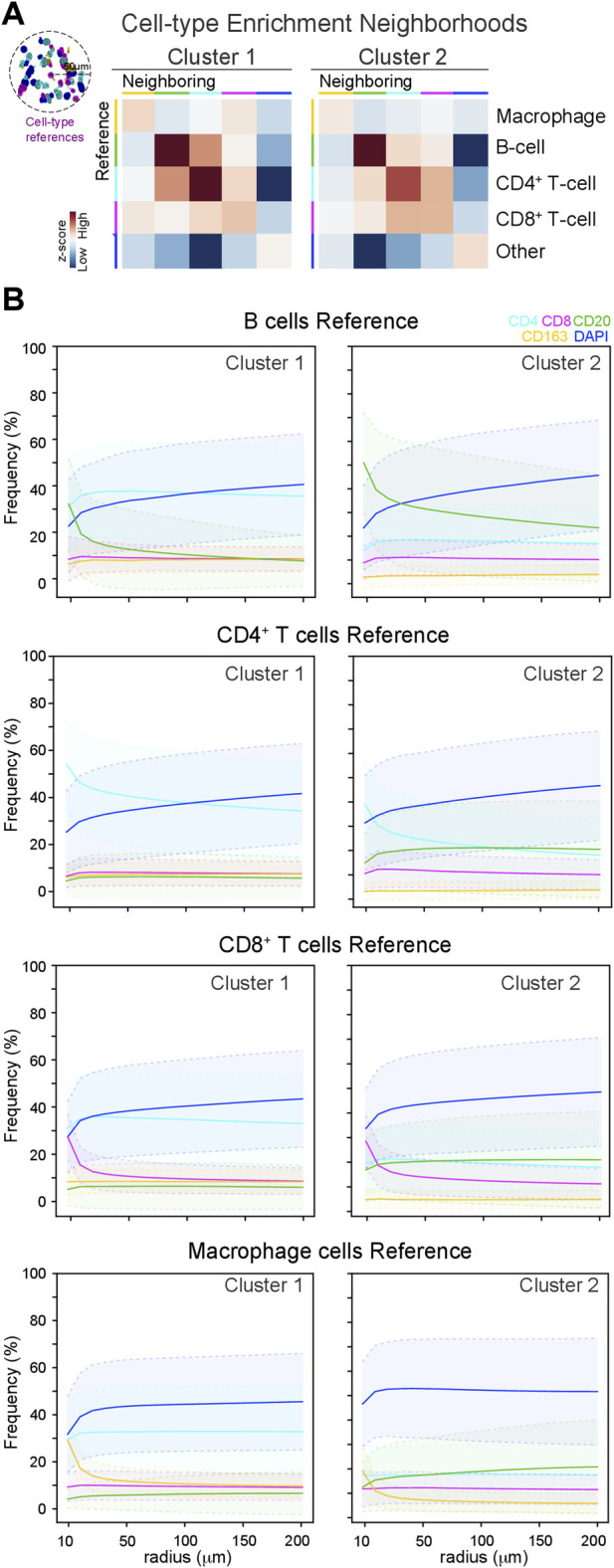
Proximity analyses of immune cell interactions. **(A)** Neighborhood enrichment analysis quantifying spatial co-occurrence patterns between immune cell types within 50 μm radius. Rows represent the reference cell types and columns represent neighboring cell types. Heatmaps display enrichment z-scores for each cell type around reference cell populations, stratified by patient cluster. Statistical significance assessed through permutation-based testing (1,000 iterations) comparing observed enrichment against null distribution. **(B)** Proximity analysis quantifying the relative fraction of each immune cell type in the neighborhood of reference cell populations across increasing radii (10–200 μm, 10 μm bins). Proximity profiles were computed at single-ROI level for each reference cell type (B cells, CD4^+^ T cells, CD8^+^ T cells, macrophages) and then averaged within clusters. Curves show mean frequencies ± standard deviations of target cell types. Shaded areas represent standard deviations.

To further examine spatial interactions between immune cells, we performed a neighborhood enrichment analysis [23]. In CL-1, B cell neighborhoods were enriched for both B cells and CD4^+^ T cells, with only limited representation of CD8^+^ T cells. By contrast, in CL-2, B cell neighborhoods retained enrichment in CD4^+^ T cells but showed reduced self-enrichment of B cells (Figure 3A). Notably, co-occurrence of CD8^+^ T cells and CD163^+^ macrophages was observed in CL-1 but absent in CL-2 (Figure 3A).To investigate these relationships across broader spatial scales, we next performed proximity analysis. Relative fractions of neighboring cell types were quantified across increasing spatial radii and aggregated across ROIs within each cluster, considering all cell types included in the analysis. As shown in Figure 3B, immune spatial organization at the invasive margin diverged between clusters. In particular, in CL-1 the immune lineages CD8, CD20 and CD163 exhibited closer spatial proximity to CD4^+^ cells, suggesting a central role for CD4^+^ T cells in local immune organization. Conversely, in CL-2 cluster, CD20^+^ B cells emerged as the main spatial hub, with all other immune subsets showing preferential proximity to CD20^+^ cells. These patterns indicate distinct spatial organization across clusters, with differences in the relative proximity of CD4^+^, macrophage and B cell populations.

### Mutational and clinical correlates of immune clusters

The study cohort included tumors arising from different anatomical sites (iCCA, pCCA, dCCA, and GBC), and cluster assignment was not associated with tumor location, with no statistically significant differences between groups (Supplementary Table 1).

We then evaluated potential associations between these immune spatial patterns and clinical or molecular tumor features. Targeted NGS was successfully performed on a subset of cases with available tumor material (n = 9/11 patients), revealing a limited number of alterations across a panel of cancer-related genes (Table 1). Within this subset, among CL-2 patients with available molecular data (3/5), all cases harbored co-occurring TP53 and ARID1A alterations (3/3), whereas no such co-occurrence was observed in CL-1 (0/6) (p = 0.0119, Fisher exact test) (Figure 2A).

**FIGURE 4:**
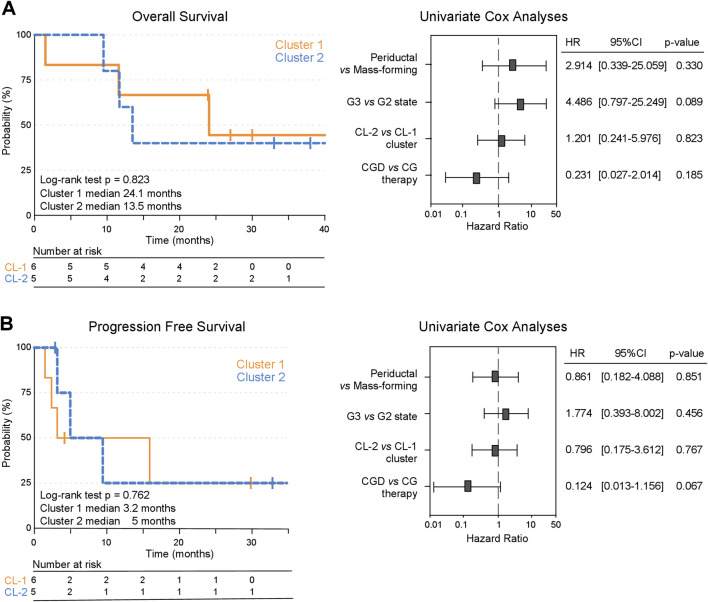
Survival analysis by immune cluster. **(A,B)** Survival outcomes stratified by immune cluster assignment. Left panels show Kaplan-Meier survival curves comparing Cluster 1 (n = 6, orange) and Cluster 2 (n = 5, blue) for **(A)** overall survival and **(B)** progression-free survival. Shaded areas represent 95% confidence intervals (CI); median survival times and log-rank test results are indicated. Right panels display forest plots for univariate Cox proportional hazards analyses for four clinical covariates (invasive pattern, therapy regimen, differentiation state and cluster assignment). Hazard ratios and 95% CI are plotted on logarithmic scale with vertical dashed line at HR = 1.

We next evaluated whether cluster assignment was associated with clinical outcome (Table 2). Kaplan–Meier analysis did not reveal statistically significant differences in OS or PFS between clusters (Figures 4A,B, left), with log-rank p values of 0.82 and 0.76, respectively. Descriptively, median OS was 24.1 months in CL-1 and 13.5 months in CL-2, while median PFS was 3.2 months and 5.0 months, respectively. However, these differences were not supported by the overall shape of the survival curves. Consistently, univariate Cox models did not identify a significant association between cluster assignment and survival outcomes (OS HR = 1.201; PFS HR = 0.796).

Additional univariate Cox regression analyses for OS and PFS, were performed across clinical variables, namely growth pattern (periductal vs. mass-forming), grading (G2 vs. G3) and treatment regimen (CG vs. CGD) (Figures 4A,B, right); none showed statistically significant associations with OS or PFS.

**FIGURE 5:**
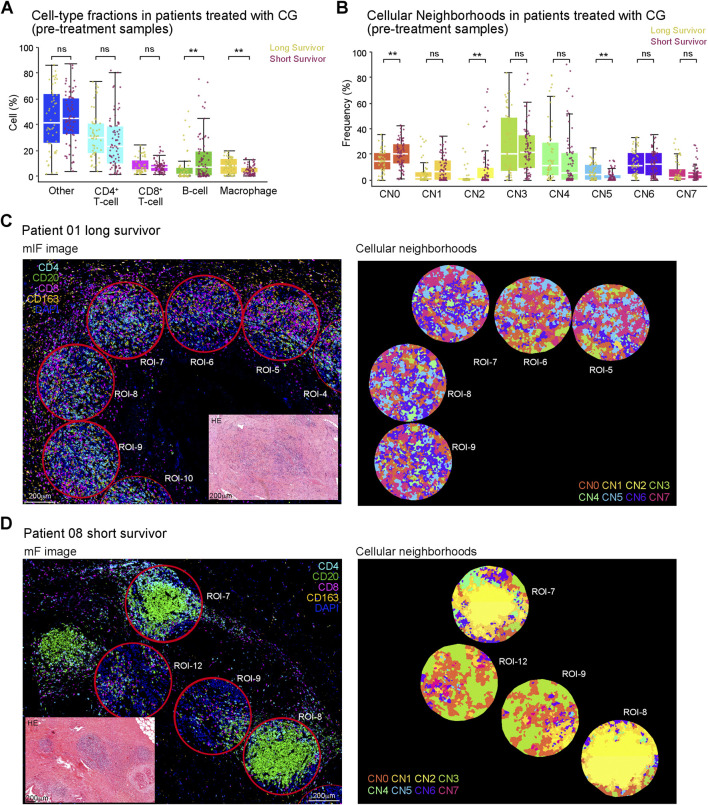
Immune correlates of survival in CG-treated patients. **(A,B)** Boxplots showing fractions in CG-treated patients stratified by survival outcome. Dots represent the relative fractions of a specific cell type **(A)** and a specific CN **(B)** within individual ROIs. Patients are grouped for long (n = 3) versus short (n = 4) survivor status based on the entire cohort median OS (23.9 months). Mann-Whitney U tests with Benjamini-Hochberg correction were used for comparisons. Asterisks indicate significance levels (ns p > 0.05 and **p ≤ 0.01). **(C,D)** Spatial visualization of cellular neighborhoods in representative CG-treated patients: Patient 01 [long survivor, **(C)**] and Patient 08 [short survivor, **(D)**]. Left panels show mIF images; right panels display CN assignments through Voronoi diagrams for selected ROIs. Scale bars are indicated in the figure.

To explore treatment-associated correlates of outcome in relation to immune phenotype, we stratified the cohort according to overall survival above or below the cohort median (23.9 months). Within the CG subgroup, this yielded numerically balanced long- and short-survival groups, allowing exploratory comparison of baseline immune features (Figures 5A,B). Long survivors showed higher fractions of CD163^+^ macrophages and lower CD20^+^ B cells at baseline, and were enriched in regions characterized by high CD163^+^ macrophage content with limited lymphoid representation (CN5) and depleted in B-cell–rich regions (CN2). The same analysis was not performed in the CGD subgroup because only one patient fell into the short-survival category.

## Discussion

In this exploratory study, we used mIF to characterize the immune architecture of BTCs, a diverse and clinically challenging group of malignancies. Using a focused 4-lineage immune panel (CD4, CD8, CD20, CD163), we retrospectively analyzed nearly 400,000 single cells across 198 tumor regions from 11 patients with advanced BTC, integrating compositional, spatial, and neighborhood-level analyses. Despite the modest sample size, our findings reveal distinct patterns of immune diversity that appear to be independent of tumor anatomical location, yet potentially informative of clinical trajectories.

One of the most interesting observations from our study is the emergence of two distinct immune clusters, one enriched in CD4^+^ T cells and CD163+ macrophages (CL-1), and the other in CD8^+^ cytotoxic T cells and CD20^+^ B cells (CL-2). These clusters did not map onto traditional BTC subtypes such as iCCA, pCCA, dCCA, or GBC, in our series. This suggests that immune contexture may represent a biologically meaningful layer of classification shared by different anatomical types of BTCs. This finding is consistent with pan-cancer evidence demonstrating that immune phenotypes, particularly those based on T cell exclusion, inflamed profiles, or myeloid predominance, often cut across tissue types [24, 25]. In the case of BTC, which has long been considered a largely immune-desert tumor, our data reveal that spatial immune phenotypes are more nuanced and may represent underappreciated axes of heterogeneity.

Beyond cell frequencies, spatial arrangement emerged as a key discriminator between immune clusters. In CL-2 tumors, immune neighborhoods rich in CD20^+^ B cell-rich regions (corresponding to CN1 and CN2) were prevalent, and B cells appeared to anchor proximity networks, often co-localizing with CD8^+^ T cells in patterns reminiscent of TLS-like arrangements. Such structures have been linked to improved responses to immunotherapy in other malignancies, including melanoma, non–small cell lung cancer, and renal cell carcinoma [26, 27]. Whether these structures in BTC represent true TLS, and whether they support or hinder antitumor immunity in this context, remains to be explored, also considering the equivocal, although only exploratory, prognostic role in our cohort. In contrast, CL-1 tumors showed a very different architecture dominated by CD4^+^ T cells and CD163+ macrophages, consistent with enrichment in CN4–CN6, which capture these spatial configurations. This could reflect a more suppressive or tolerogenic TIME, possibly driven by T helper-macrophage crosstalk. CD163^+^ macrophages have been implicated in immune suppression and tumor progression in BTC and other gastrointestinal tumors [28–30].

Although in our cohort patients enriched in CD163^+^ and depleted in CD20^+^ cells had numerically longer mOS, they were enriched also in CD4^+^ T helper lymphocyte, that have been recently showed to be prognostically favorable when FOXP3 negative [31]. Moreover, the presence of B cells may not equate to an effective antitumor response; in fact, B cells may contribute to dysfunctional or regulatory immune niches depending on their subtype [32]. Second, the spatial exclusion of CD8^+^ T cells from tumor nests, a pattern we did not directly quantify here but that may be suggested by spatial proximity data, might suggest ineffective infiltration despite high abundance.

Alternatively, the association of CD163-rich, B-cell-poor TIME with better survival in CG-treated patients might reflect a distinct immune state that is more sensitive to chemotherapy alone, perhaps due to macrophage-mediated phagocytosis or modulation of the stromal microenvironment. These hypotheses remain speculative but highlight the need to contextualize immune phenotypes not only by cell presence, but by function, localization, and interaction.

While exploratory, the observation that CL-2 patients were more likely to harbor co-occurring TP53 and ARID1A mutations is noteworthy, as data on the co-occurrence of these alterations remain limited and poorly described in the literature. Both genes have been linked to increased tumor mutational burden, altered chromatin states, and immune activation in several cancers [33, 34]. ARID1A loss, in particular, has been associated with enhanced neoantigen presentation and interferon signaling [35, 36], while TP53 mutations have been implicated in genomic instability, dysregulated immune signaling, and context-dependent modulation of tumor-immune interactions.

The co-occurrence of these alterations may therefore reflect a convergent mechanism in which chromatin remodeling defects and impaired genomic surveillance jointly promote an immunogenic tumor state. We speculate that this combination could contribute to the establishment of distinct immune niches, potentially characterized by increased antigenicity but also adaptive immune resistance. These findings suggest that specific genomic events may shape immune niches, and that integrating spatial profiling with genomic data may reveal co-dependencies or vulnerabilities relevant to treatment stratification.

The recent incorporation of durvalumab into first-line treatment for BTC has marked a new era of chemo-immunotherapy in this disease. However, responses remain heterogeneous, and biomarkers are urgently needed. Our study suggests that baseline spatial immune states, particularly the presence or absence of lymphoid-rich or macrophage-dominated neighborhoods, may correlate with treatment outcome, yet possibly differently in CG and CGD cohorts. Given the small number of patients in this study, no preliminary conclusions can be drawn regarding immune predictors of response to immunotherapy. However, the divergence in TIME composition suggests a rationale for future prospective studies to stratify patients based on immune spatial features, perhaps identifying subsets more likely to benefit from checkpoint blockade or requiring alternative strategies (e.g., macrophage modulation, B cell targeting).

This study is subject to several limitations. The small sample size and retrospective nature preclude formal statistical testing or definitive prognostic claims. The immune panel was limited to four markers, and deeper phenotyping (e.g., regulatory T cells, dendritic cells, immune checkpoints) would provide a more granular view. Functional readouts such as gene expression, T cell clonality, may further enrich the immune characterization of the TIME. Furthermore, while we identified immune neighborhoods, we did not directly map TLS using canonical markers (e.g., PNAd, CXCL13, CD21) [37], which will be important in follow-up studies. Lastly, the presence of a single patient treated with CGD classified as short surviving allows descriptive comparison based on survival only in the CG cohort. Nonetheless, this work establishes a foundation for spatially informed immune classification in BTC. It supports the concept that TIME heterogeneity is biologically meaningful and may yield new insights beyond conventional histopathological or molecular subtyping. Future efforts should aim to integrate spatial data with genomic, transcriptomic, and clinical variables in larger, prospective cohorts, ideally embedded within immunotherapy trials, to define actionable immune states and guide rational combination strategies.

In summary, spatial immune profiling of BTC using a focused 4-marker panel revealed two major immune phenotypes that appear to be independent of tumor location, yet distinct in composition, architecture, and potentially in clinical trajectory. Although survival differences between CL-1 and CL-2 did not reach statistical significance, the observed trend warrants validation in larger cohorts. These findings highlight the importance of immune topology in understanding BTC biology and treatment response. As the therapeutic landscape of BTC evolves, spatially resolved immune biomarkers may play an essential role in personalizing care for this highly heterogeneous disease.

## Data Availability

The datasets presented in this study can be found in online repositories. The names of the repository/repositories and accession number(s) can be found below: https://github.com/ComputationalCancerImmunotherapyLab/SpatialProteomicCholangiocarcinoma.
