# Supplementary figures and images for: Proof-of-principle 4-marker spatial profiling reveals distinct, location-independent immune clusters in biliary tract cancers
Source: Pathol Oncol Res. 2026 May 28;32:1612389. doi: 10.3389/pore.2026.1612389 (PMC13253487; doi:10.3389/pore.2026.1612389)

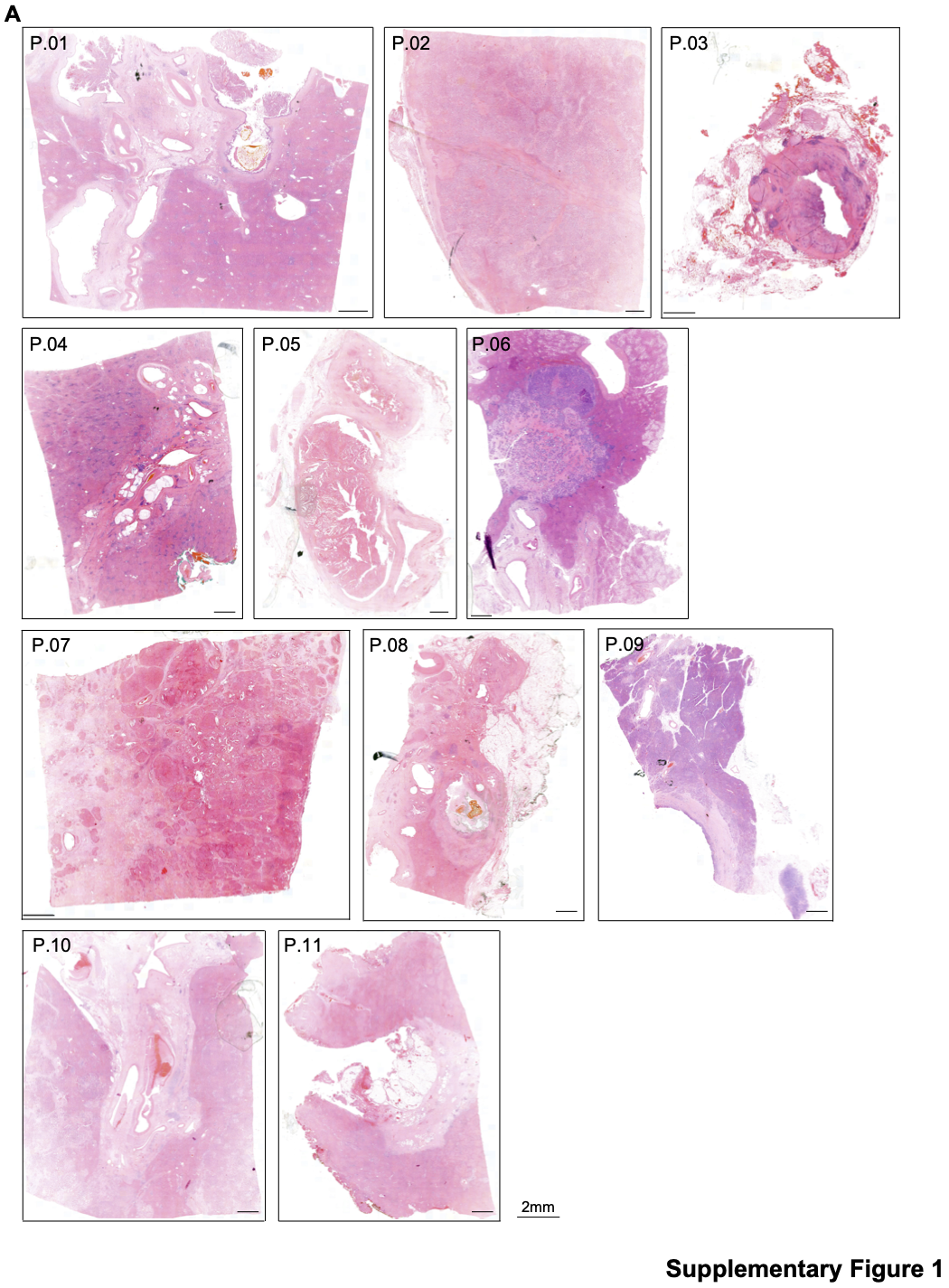

Supplement: Supplementary file 1 [file Image1.tiff]

A

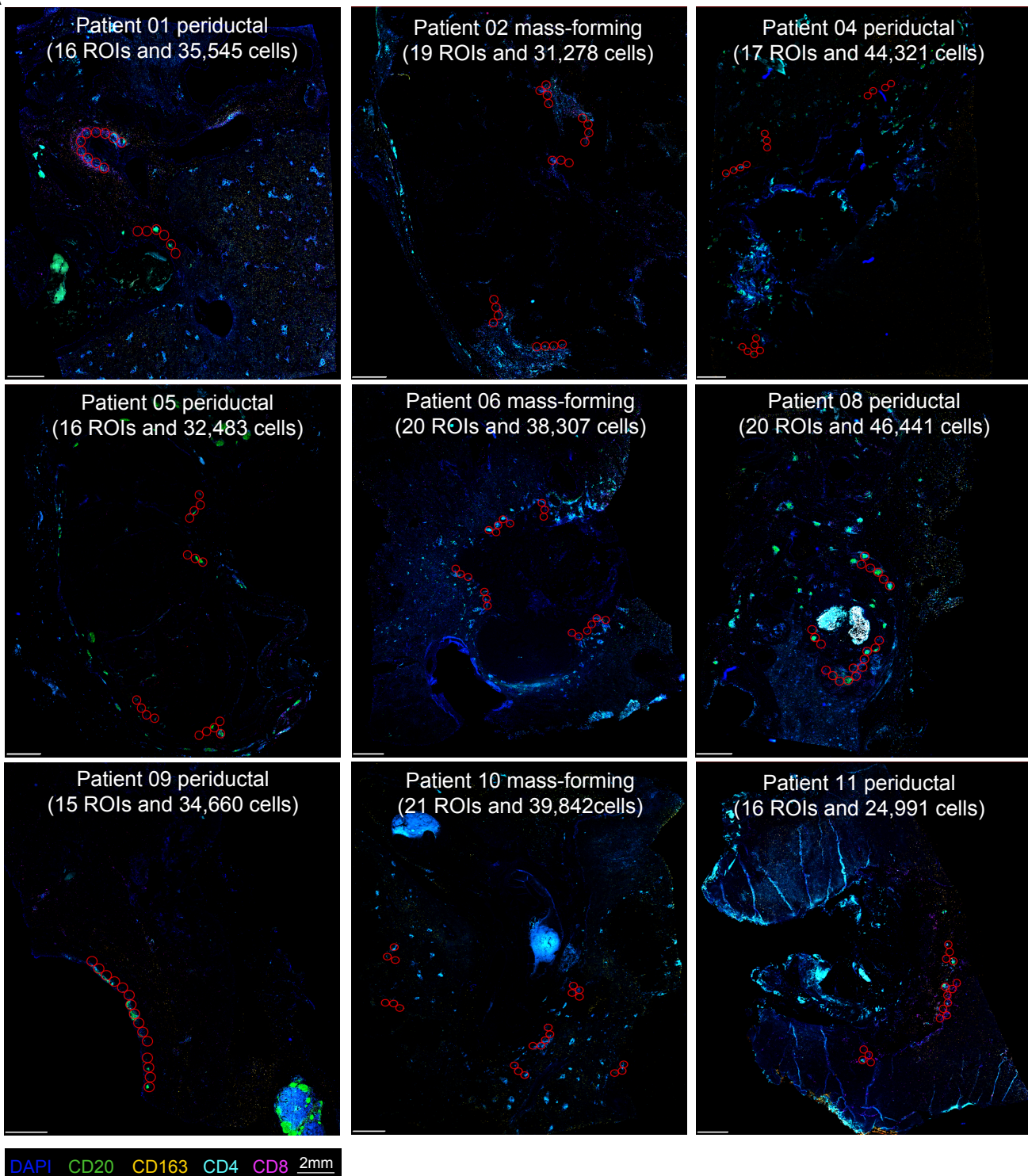

Supplementary Figure 2

Supplement: Supplementary file 2 [file Image2.pdf]
