# Supplementary material for: Proof-of-principle 4-marker spatial profiling reveals distinct, location-independent immune clusters in biliary tract cancers
Source: Pathol Oncol Res. 2026 May 28;32:1612389. doi: 10.3389/pore.2026.1612389 (PMC13253487; doi:10.3389/pore.2026.1612389)
Supplement: Supplementary file 3 [file Image3.pdf]

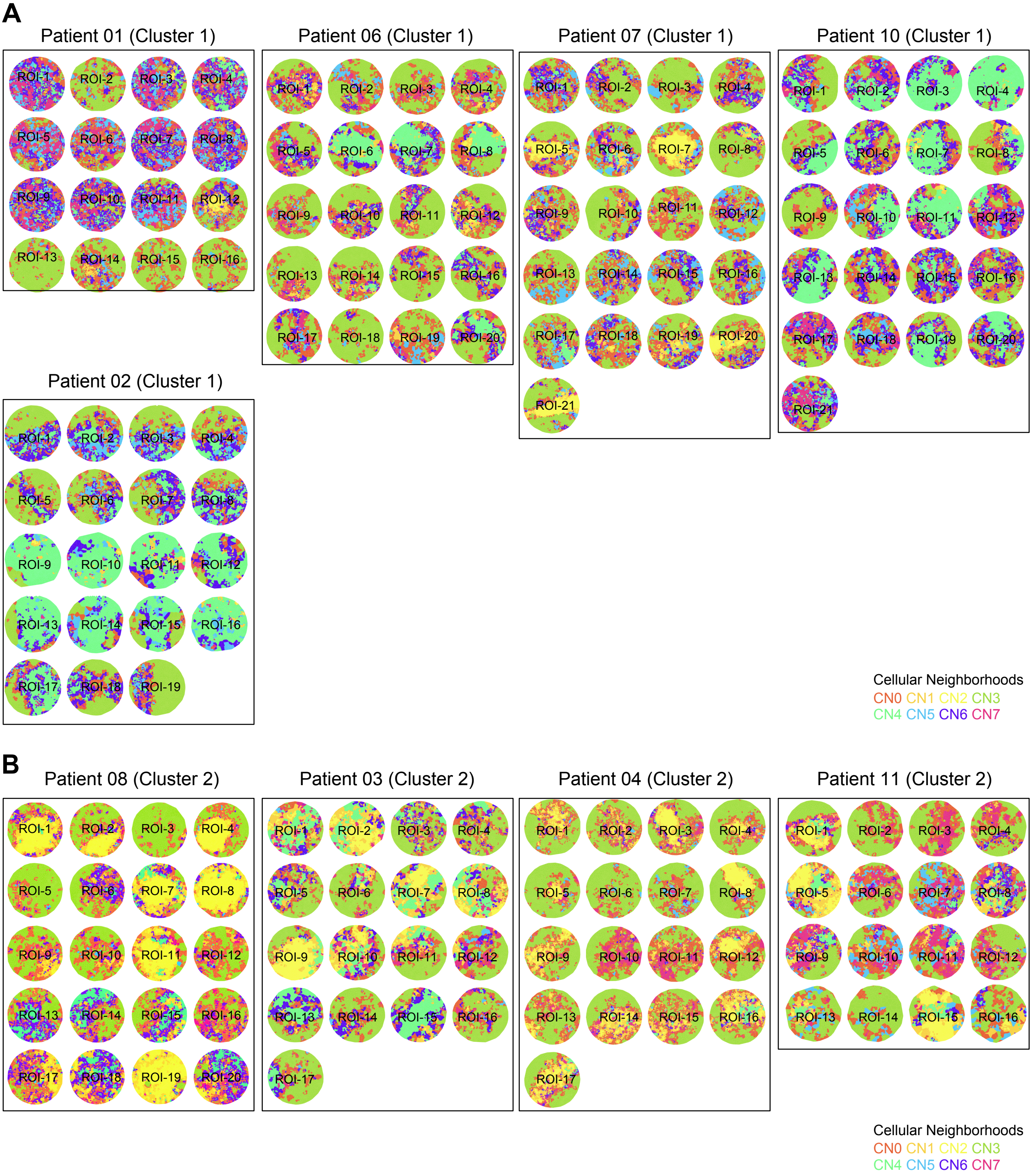

Supplementary Figure 3
