# Supplementary material for: Proof-of-principle 4-marker spatial profiling reveals distinct, location-independent immune clusters in biliary tract cancers
Source: Pathol Oncol Res. 2026 May 28;32:1612389. doi: 10.3389/pore.2026.1612389 (PMC13253487; doi:10.3389/pore.2026.1612389)
Supplement: Supplementary file 4 [file DataSheet1.pdf]

Supplementary Material

Supplementary Figure 1. Histological imaging. Hematoxylin and eosin (H&E) stained entire tissue sections from patients in the study cohort, showing tumor morphology and invasive pattern. Scale bars are indicated in the figure.

Supplementary Figure 2. mIF imaging. Cell nuclei (DAPI) and CD4, CD8, CD20, and CD163 signals are displayed in pseudocolors. For each patient the invasive pattern classification and the total number of ROIs and cells characterized are indicated. Scale bars are indicated in the figure.

Supplementary Figure 3. Comprehensive visualization of cellular neighborhood (CN) assignments across all patients. (**A-B**) Cellular neighborhoods visualization across all ROIs from patients in the study cohort through Voronoi diagrams colored by CN assignment.

Supplementary Table S1. Aggregated characteristics of the study population and of patient clusters.

Characteristics	Overall	Cluster 1	Cluster 2	p value
**Sex**				
Male	8 (72.7%)	3 (50%)	5 (100%)	Fisher test, p = 0.1818
Female	3 (27.3%)	3 (50%)	0 (0%)	
**Age**				
>70	5 (45.5%)	3 (50%)	2 (40%)	Fisher test, p = 1.000
≤70	6 (54.5%)	3 (50%)	3 (60%)	
**Tumor location**				
iCCA	4 (36.4%)	3 (50.0%)	1 (20.0%)	Chi-square, p = 0.5238
pCCA	5 (45.4%)	2 (33.3%)	3 (60%)	
dCCA	1 (9.1%)	1 (16.7%)	0 (0.0%)	
GBC	1 (9.1%)	0 (0.0%)	1 (20.0%)	
**Somatic mutations**				
TP53	4 (44.4%)	1 (16.7%)	3 (100.0%)	Fisher test p = 0.0476
ARID1A	4 (44.4%)	1 (16.7%)	3 (100.0%)	Fisher test p = 0.0476
TP53::ARID1A	3 (33.3%)	0 (0.0%)	3 (100.0%)	Fisher test p = 0.0119
**Treatment for advanced disease**				
CG	7 (63.6%)	4 (66.6%)	3 (60.0%)	Fisher test, p = 1.000
CGD	4 (36.4%)	2 (33.4%)	2 (40.0%)	
**Responder**				
R	3 (27.3%)	4 (66.7%)	1 (20.0%)	Fisher test, p = 0.2424
NR	8 (72.7%)	2 (33.3%)	4 (80.0%)	
**Best RECIST 1.1 response**				
CR	1 (9.1%)	0 (0.0%)	1 (20.0%)	Chi-square, p = 0.4857
PR	2 (18.2%)	2 (33.3%)	0 (0.0%)	
SD	4 (36.4%)	1 (16.7%)	3 (60.0%)	
PD	4 (36.4%)	3 (50.0%)	1 (20.0%)	

p-values were calculated using Fisher's exact test or Chi-square test as appropriate.

Abbreviations: iCCA, intrahepatic cholangiocarcinoma; pCCA, perihilar cholangiocarcinoma; dCCA, distal cholangiocarcinoma; GBC, gallbladder cancer; CG, cisplatin plus gemcitabine; CGD, cisplatin plus gemcitabine plus durvalumab; RECIST, Response Evaluation Criteria in Solid Tumors; CR, complete response; PR, partial response; SD, stable disease; PD, progressive disease; R, responder; NR, non-responder.
